# Bridging the Gap: A narrative review of osteoporosis disability, adipokines, and the role of AI in postmenopausal women

**DOI:** 10.12669/pjms.40.7.9072

**Published:** 2024-08

**Authors:** Saba Tariq, Sohail Jabbar, Awais Ahmad, Sundus Tariq

**Affiliations:** 1 Saba Tariq, Department of Pharmacology & Therapeutics, University Medical and Dental College, The University of Faisalabad, Post-doctoral Fellow, University of Birmingham, England, UK; 2 Sohail Jabbar, Department of Computer Science, College of Computer and Information Sciences, Imam Mohammad Ibn Saud Islamic University (IMSIU), Riyadh, Saudi Arabia; 3 Awais Ahmad, Information Systems Department, College of Computer and Information Sciences, Imam Mohammad Ibn Saud Islamic University (IMSIU), Riyadh, Saudi Arabia; 4 Sundus Tariq, Department of Physiology, International School of Medicine, Istanbul Medipol University, Research Institute for Health, Sciences and Technologies (SABITA), Turkey

**Keywords:** Osteoporosis, Adipokines, Artificial intelligence, BMD

## Abstract

Osteoporosis is a global health concern characterized by reduced bone density and compromised bone quality, resulting in an increased risk of fractures, particularly in postmenopausal women. The assessment of bone mineral density (BMD) plays a pivotal role in diagnosing osteoporosis, as it accounts for approximately 70% of overall bone strength. The World Health Organization (WHO) has endorsed BMD measurement as a reliable method for diagnosing this condition. In Pakistan, the incidence of bone fractures is on the rise, largely attributable to an aging population and a range of contributing factors. Understanding the global and local prevalence of osteoporosis, its impact on morbidity and mortality, and the contributing factors is vital for developing effective preventive and therapeutic strategies.

The role of adipokines, including chemerin, vaspin, and omentin-1, in bone metabolism is an emerging area of investigation. These adipokines play diverse roles in physiology, ranging from inflammation and metabolic regulation to cardiovascular health. Understanding their potential impact on bone health is a topic of ongoing research. The intricate relationship between bone density, bone quality, and overall bone strength is central to understanding the diagnosis and management of osteoporosis. Current innovation in machine learning and predictive model can bring revolution in the field of bone health and osteoporosis. Early identification of people with osteoporosis or risk of fracture through machine learning can prevent disability and improve the quality of life.

## INTRODUCTION

**Fig.1:**
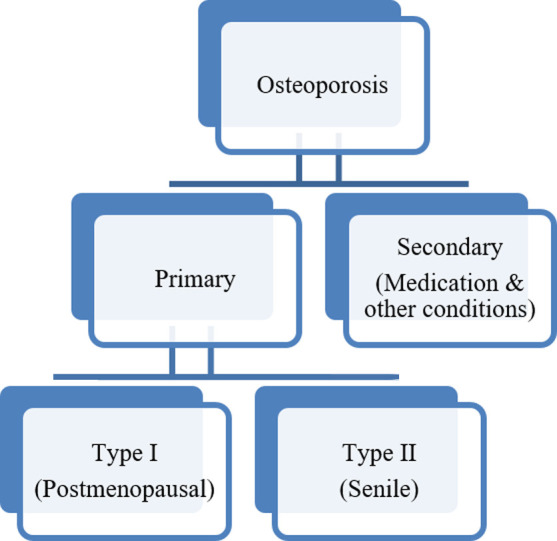
Types of Osteoporosis.

Bone density and bone quality are combined to form bone strength. Measuring BMD is an important method to measure seventy percent of bone strength and is used to diagnose osteoporosis. This method is also acceptable by World Health Organization (WHO).^1^ A large number of people are suffering from osteoporosis worldwide, and WHO consider it as a one of the serious global non-communicable disease. Osteoporosis is a chronic disorder and one of the common cause of fractures in postmenopausal females Among these fractures, the most serious is hip fracture, which is the main cause of increased morbidity and mortality. The increase in morbidity is associated with social as well as economic burden.^1^ Osteoporosis is divided in to two main types (Fig.1).

An epidemiological statistic shows that among all the other commonly encountered age matched disorders “osteoporosis” is considered as number one in women and number two in men. Osteoporosis is a health issue of main concern among people that is expanded over time to results in 8.1 million bone fractures (78 % women, 22 % men) in a timeframe from 2010 to 2050.^2^

Osteoporosis is regarded as the most common cause of fractures in USA and in a recently conducted study 27.7% patients were found to have bone fractures due to osteoporosis after the 5^th^ decade of age, and among them 88 (57.5%) patients got fractures after minor trauma.^3^ United States (US) Medicaid, affirms the statistics in the timeframe of 2002-2015 for females 65 years of age suggested that hip fracture ratio for age matched individuals for the three consecutive years 2013, 2014, and 2015 were much advanced than calculated, as a result of which there is an approximate rise of number of patients having hip fracture i.e. exceeding 11,000.^4^ As there is exponential increase in osteoporotic fracture annually, the amount spent to take care of these fractures surpass those of many other major diseases, including breast cancer, heart disease, and stroke. Among patients with hip fractures, the presence of frailty, as quantified by the Hospital Frailty Risk Score (HFRS), is closely linked to heightened morbidity, mortality, and escalated healthcare costs. Notably, individuals classified in the highest HFRS category exhibit a pronounced increase in median total hospitalization expenses, reaching SGD $22,432 (4,665,856 PKR).^5^

In Pakistan, the frequency of bone fractures is increasing because of increasing age of the population and many other factors are implicated in this process such as frequency of osteoporosis being more common in poor, illiterate, multiparous and sedentary women.^6^ Another study found high pervasiveness of osteoporosis (13.26%) and osteopenia (22.98%) among Pakistani population.^7^ It has been reported that in Faisalabad, the prevalence of osteopenia and osteoporosis was 29.8%, 27.2%, respectively, while 43% subjects had normal BMD.^8^

### Adipokines

Adipokines released by white adipose tissue (WAT) and bone marrow adipose tissue (BMAT) affect osteoblast and osteoclast survival and function in endocrine and paracrine ways. Understanding the intricate interactions between adipocytes and bone is essential since osteoporosis is linked to an increase in marrow fat. Novel adipokines such as chemerin, vaspin, omentin-1 and Osteoprotegerin (OPG) have shown bone-protective and osteoanabolic properties that could be translated into therapeutic targets for the treatment of osteoporosis.^9^

### Chemerin

Chemerin, a versatile novel adipokine is a 14-kDa protein is secreted by adipose tissue and liver in an inactive form, which is latter converted into an activated form by cleavage of C-terminus by serine proteases 10. Chemerin and its receptors have been widely studied and scientists have been able to discover its role in different metabolic and neuroendocrine functions.

### Chemerin Receptors

Chemerin mainly performs its function by acting on two types of receptors. One of the receptor is designated as Chemokine-Like Receptor 1 also known as Chemerin Receptor- 1 (CMKLR1). The other receptor also known as chemerin receptor 2 is a G Protein–Coupled Receptor-1 (GPR1). The chemokine like receptor-1 is present in abundant amount in macrophages, monocytes and dendritic cell. Whereas other tissues of the body such as adipose tissue, lymph nodes and spleen also shows high expressions of these receptors.^11^

### Physiological Role of Chemerin

Chemerin receptor-1 can initiate an immune response. This has been shown by the expression of these receptors on macrophages and dendritic cells. The pro-inflammatory property of chemerin causes reincarnation of dendritic cells and macrophages in human.^12^ Chemerin plays an important role in adipogenesis as it is secreted by adipose tissue. Knockdown of chemerin in both human and mouse not only results in inhibition of adipogenesis but also leads to inhibition of human bone marrow stromal cells which have the ability to differentiate in to adipocytes.^11^

### Chemerin and bones

Adipocytes are involved in bone remodeling. They influence differentiation and function of two important bone cells osteoblasts and osteoclasts. It is important to maintain balance between bone formation and resorption in order to maintain skeletal system strength and integrity. Adipocytes play a main role in bone homeostasis as well as in different disorders of bone. These adipocytes and osteoblasts cells are derived from same mesenchymal stem cells precursors.^13^

In a study conducted in db/db mice, it was observed that chemerin is not only involved in bone loss but also the administration of chemerin receptor antagonist CCX832 results in inhibition of bone loss. They concluded that in vitro chemerin has the ability to increase osteoclastic activity.^13^ In contrast to this study, a recently conducted study found that CMKLR1 knockout mouse has lower trabecular bone mass. Scientists were of the opinion that androgen the hormone responsible for bone homeostasis in males is dependent on CMKLR1 for its proper function and loss of these receptors results in lower bone mineral density.^14^,^15^

The controversial effect of chemerin led to more investigation of this peptide and exploration of its role in human bones. In one study conducted in 8826 adults aged 20 to 79 years in general population of Germany researchers determined the effect of chemerin on bone quality measured by Qualitative Ultrasound Scan with respect to body mass index and they found that chemerin is inversely associated with bone quality in obese females and positively associated with fracture risk. However, they could not found any siginificant association of chemerin with bone in lean individuals.^16^ A study in Chinese woman demonstrated that serum chemerin had an inverse relationship with BMD especially in elderly postmenopausal females.^17^ Another study observed no association of chemerin with bone mineral density in postmenopausal osteoporotic and non-osteoporotic females.^18^

### Vaspin

Vaspin, a serine protease inhibitor is derived from visceral adipose tissue and is also known as SERPINA12 according to serpin nomenclature.^19^ Its structure is highly specific and contains a protease recognition sequence, which is present on the top of a reactive central loop also known as RCL.^20^

Investigations are being done to discover different role of vaspin in human body. Clinical and exploratory studies have proposed the extraordinary and promising multifaceted capacities of vaspin activity in the fat, as well as in a wide range of cells, tissues and organs.^19^

### Vaspin Mechanism

Vaspin works by securing cells and tissues from proinflammatory conditions. This defensive or neutralizing capacity, regardless of whether in adipocytes, vascular, skin or bone cells, appears to be executed by means of both, the direction of protease action and the connection with cell surface receptors, for example, GRP78. It is KLK7 from the kallikrein family (KLKs) to be repressed by vaspin by means of the traditional serpin system. However, the correct fundamental instruments of signal transduction for some of the revealed impacts of vaspin warrants into more research and is expected to dismember the contributing pathways.^19^,^20^

### Physiological Role of Vaspin

Vaspin, as a newly found adipocytokine, has insulin sensitizing effects that can modulate obesity. In a recent study conducted in rats it was found that vaspin level would be a better analytic and diagnostic marker with regards to the insulin resistance.^21^ This insulin sensitizing action of vaspin depends upon enhancing GLUT4 expression and receptors translocation with more established human skeletal muscle to insulin-intervened glucose take-up.^22^ Developing proof has also suggested that vaspin is effectively engaged with the advancement of atherosclerotic cardiovascular diseases and serum vaspin levels were emphatically identified with carotid atherosclerosis, and is a significant prognostic marker in acute myocardial infarction.^14^

### Vaspin and bone

Studies assessing the connection among vaspin and bone mineral density (BMD) have acquired different results. The role of vaspin in bone homeostasis has been researched in vitro examinations showing two-sided consequences for both, bone forming osteoblasts and bone-resorbing osteoclasts. In vitro studies demonstrated that vaspin, in a dose-dependent manner, promoted osteogenic differentiation and ALP activity in rat primary OBs. Additionally, vaspin upregulated mRNA expression of osteogenesis-related genes Runx2, Osx, and Colla1, along with protein expression of Runx2, Smad2/3, and p-Smad2/3 signaling pathways.^23^

**Table-I:** Properties of adipokines relevant in bone diseases. 10,11,16-18,19,24-27,33-35,37-40

Adipokines	Source	Structure	Receptors	Signaling	Correlation with BMD
Chemerin	Adipose tissue and liver	14-kDa protein	Chemokine-Like Receptor 1 (CMKLR1). Chemerin receptor 2, a G Protein–Coupled Receptor 1 (GPR1)	Initiate an immune response	Controversial
Vaspin	Visceral adipose tissue	Serine protease inhibitor belongs to SERPINA12 family. Contains nine alpha helices and three beta sheets	Serine protease receptors	Action via cell surface receptors, for example, GRP78.	Positive
Omentin-1	Visceral adipose tissue	Consist of 313 aminoacids	No particular receptors	PI3K/Akt signaling pathway	Controversial
OPG	Osteoblasts and osteogenic stromal stem cells	Dissoluble glycoprotein which exists in either two forms a 60-kDa polymer or a 120-kDa.	Cytokine receptor of the tumour necrosis factor (TNF) receptor superfamily	Bind with RANK Ligand and is a natural inhibitor of RANKL	Positive

Another study was conducted in diabetic osteoporotic and non-osteoporotic group observed that serum vaspin levels were lower in diabetic group with osteoporosis as compare to diabetic group without osteoporosis. Low levels of vaspin are also involved in the advancement of diabetic osteoporosis through different ways that impact the bone metabolism.^24^

In addition to these studies vaspin was also investigated in other bone diseases. In patients with psoriatic arthritis it was observed that vaspin levels are significantly altered in control and patients with psoriatic arthritis suggested its involvement in bone related diseases.^25^ These contradictory studies required further investigation to elaborate vaspin role in bone metabolism.

### Omentin-1

Mammalian fat is composed of white fat (subcutaneous and visceral fat) and brown fat. The visceral fat is responsible for secretion of variety of adipocytokines which are involved in various metabolic functions.^26^ Omentin-1 is a novel adipokine consisting of 313 aminoacids is secreted from visceral adipose tissue.^27^ It has been observed that Human Omentin-1 expression can be increased with certain factors such as dexamethasone have the ability to increase its expression whereas certain other endogenous substances can decrease its expression such as insulin.^28^

### Omentin-1 Receptors

As till now no particular receptors of omentin-1 have been identified and it is thought that omentin-1 is basically not working through receptors but the signaling pathway of omentin could be non-protein and omentin-1 is performing its function by binding to carbohydrates and glycolipids. However, it is particularly essential to explain the putative receptor, which gives an examination of its agonist as another drug target and new treatment modalities can be found.^29^

### Pathophysiological role of Omentin-1

Different physiological roles of omentin have been identified out and it has been observed that Omentin-1 has a positive influence on energy homeostasis and glucose metabolism. Along with these effects it has a cardiovascular protective effect as it possesses both anti-inflammatory and antioxidant properties.^30^

It has been observed that circulating levels of Omentin-1 are decreased in diseases related to intestine such as inflammatory bowel disease.^31^ Omentin-1 has been investigated as a possible prognostic index for coronary artery disease and heart failure. Increase levels of omentin-1 are also associated with increase cardiovascular events and mortality.^32^

### Omentin-1 and Bone

It has been proposed that omentin-1 increase osteoblast proliferation through a signaling mechanism involve in cell cycle regulation known as PI3K/Akt signaling pathway.^33^ A study showed that omentin-1 levels are increased in postmenopausal osteoporotic females as a result of compensation to bone loss that occurs after menopause.^34^ Omentin-1 may apply a negative impact on bone mass through the regulation of osteoblast differentiation.^35^ These results open the horizons for further investigation to rule out the potential effect of omentin-1 on bone homeostasis.

### Osteoprotegerin (OPG)

Bone remodeling is one of the homeostasis mechanism human body encounters throughout the skeletal system life. Correspondingly two major mechanisms involving RANKL/RANK/OPG and Wnt pathways produce signals that have both local and systemic effects. Bone remodelling cycle is a highly controlled regulatory system maintaining the balance between bone resorption and bone formation and it involves these two key pathways. Thus indicating that these pathways may have role for diagnostic and therapeutic interventions in bone diseases like osteoporosis.^36^

### Pathophysiological Role of OPG

OPG also known as osteoclastogenesis inhibitory factor, OCIF or TNF superfamily member 11B (TNFRSF11B) gene which encodes a receptor for chemokines called cytokine receptor of the tumour necrosis factor (TNF) receptor superfamily. Osteoprotegerin is a dissoluble glycoprotein which exists in either two forms a 60-kDa polymer having the same compound or a 120-kDa polymer having the compounds of two different types connected by S-S or two sulphide groups bridged by covalent bonds.^37^ OPG is also identified as proinflammatory molecule and is associated with angiogenesis and vasculogenesis and is also involved in tumour metastasis and growth.^38^

### Osteoprotegerin and Bone

Osteoblasts are bone forming cell and Osteoclast causes bone resorption. RANK is demonstrated on the surface of osteoclast and RANK Ligand attaches with RANK in order to increase osteoclast formation, function and survival. OPG, a protein secreted by Osteoblast have the ability to bind with RANK Ligand and is a natural inhibitor of RANKL. In premenopausal females there is a balance however, in postmenopausal females decrease in estrogen leads to increase expression of RANKL which then by passes OPG and leads to increase binding with RANK causing increase in osteoclast function and ultimately increase in bone resorption with eventual osteoporosis.^39^ The role of OPG as a biomarker in patients with osteoporosis is under consideration. OPG is a self-supporting indicator of hip bone fracture in women hospitalized for fragility fractures and it has been declared as a valid biomarker to diagnose females with low BMD.^40^

### Integration of AI into bone metabolism and Adipokines

In order to prevent bone fractures due to any illness or as a part of normal ageing process of the population, the knowledge about 3D bone micro architecture is required. When it comes to bones the micro architecture plays a role, in conditions like osteoporosis, osteoarthritis and predicting fractures. Nowadays we have techniques that allow us to study objects at a scale and gather information about each element that makes up the materials structure of bones. One such technique is Line Skeleton Graph Analysis (LSGA) which examines bone micro architecture at a level while providing insights, about bone health. Similarly, Machine learning (ML) algorithms have gained in popularity as a result of their modelling flexibility and capacity to identify more intricate correlations between input variables and output data, enhancing prediction. ML algorithms have been used in a number of medical fields with growing clinical utility and appropriateness. 41

Osteoporosis can be predicted using ML techniques based on clinical risk factors. In terms of performance, these models have excelled above traditional approaches, but majority of them have research concentrated on postmenopausal and older women who had a high risk of osteoporosis.^41^,^42^ Since ML methods for osteoporosis research are still in its infancy, there is currently no established route for clinical embedding. It is crucial that the technique used in ML studies should be published in sufficient detail. The performance of models in attempts to quantify fracture risk prediction was typically assessed using Area under Curve (AUC).^43^

Fracture risk assessment tool (FRAX), the currently popular model for evaluating 10-year fracture risk prediction, with an AUC is between 0.74 and 0.79.^43^ However, single performance indicators, with AUC, are insufficient, though, to suggest models for clinical use. To match the expected likelihood of the event with the actual occurrence of the event, a calibration step is required. Predicting the likelihood of osteoporotic fractures is also influenced by a number of variables, the majority of which are time-dependent. Recurrent neural networks are one example of ML models that use temporal sequences of data to predict fracture risk. Although machine learning (ML) is an exciting approach that has the potential to advance research in the field of osteoporosis, this enthusiasm needs to be restrained by the possibility of hidden biases and the requirement that researchers gain experience in this new subject in order to avoid the numerous dangers.^44^

## CONCLUSION

In order to address problems across the spectrum of osteoporosis therapy, this review has put forward an overview of current AI-based modalities. In the field of osteoporosis, the use of scans for opportunistic fracture detection or osteoporosis diagnosis has emerged as a potential technique for osteoporosis screening and a significant advancement for machine learning. The complex interplay of these factors in bone health underscores the need for continued research to understand the multifaceted nature of osteoporosis and develop effective preventive and therapeutic strategies.

### Authors’ Contribution:

**ST**: Plan the review, literature search, manuscript writing.

**SJ**: Review and final approval of the manuscript.

**AA**: Literature search, identified relevant studies, and synthesized the literature.

**ST**: critically reviewed and evaluated the literature, offering guidance and expertise.

All authors are responsible and accountable for the accuracy and integrity of the work.
